# Medicinal Use of Cannabis in Children and Pregnant Women

**DOI:** 10.5041/RMMJ.10382

**Published:** 2020-01-30

**Authors:** Gideon Koren, Rana Cohen

**Affiliations:** 1 Adelson Faculty of Medicine, Clinical Pharmacology Program, Ariel University, Ariel, Israel; 2 Motherisk Israel Program, Yitzhak Shamir Medical Center, Be’er Ya’akov, Israel

**Keywords:** Autism, cannabidiol, cannabis, children, fetal alcohol spectrum disorder, tetrahydrocannabinol

## Abstract

The increasing medicinal use of cannabis during recent years has largely overlooked children and pregnant women due to litigious and ethical concerns. However, over the last few years medicine has observed increasing numbers of children treated with cannabis for autism spectrum disorder (ASD) and fetal alcohol spectrum disorder (FASD), and pregnant women treated for hyperemesis gravidarum (HG). This review provides an account of major findings discovered through this research. Specifically, cannabis may offer therapeutic advantages to behavioral symptoms of autism spectrum disorder and fetal alcohol spectrum disorder, and to the severe nausea and vomiting in hyperemesis gravidarum. The use of medical cannabis in children and pregnant women should be further discussed and researched in this patient population.

## INTRODUCTION

The increasing medicinal use of cannabis during recent years has largely overlooked children and pregnant women due to litigious and ethical concerns.^1^ Unlike with medicinal drugs which have clear chemical, pharmacokinetic, and pharmacodynamic properties, one cannot be sure how much cannabis is delivered, by which products, because different plants, and different parts of the plant, exhibit large variability in the amounts of the different active cannabis moieties. The fears of causing harm to the child or unborn baby are well recognized, leading the medical community to be extremely careful before using cannabis.

However, outside the realm of well researched medical practice, pregnant women and parents with special-needs children often feel that their medical needs and those of their children are not met with conventional medicine. This has led an increasing number of parents to take the initiative and offer cannabis to their loved ones. While we, as medical researchers, have not been active participants in this quest, can we simply ignore it and not learn from it once it comes to our attention, often with our own patients?

Based in a developmental clinical pharmacology program in Shamir Hospital, Asaf Harofe, Israel, we have had a long interest in identifying new therapeutic modalities for children with special needs. Over the last few years we have become active observers of the increasing number of children treated with cannabis for autism spectrum disorder (ASD) and fetal alcohol spectrum disorder (FASD), and pregnant women treated for hyperemesis gravidarum (HG). This narrative review provides an account of our main findings from these preliminary observations, supported by searches of the PubMed, Embase^®^, and Cochrane databases up to July 2019.

## EFFECTIVENESS OF MEDICAL CANNABIS

### Medical Cannabidiol in Children with Autism Spectrum Disorder

Parents of children with ASD often struggle in an endless search for treatment modalities to alleviate severe symptoms such as aggression, self-harm, restlessness, sleep problems, hyperactivity, anxiety, and other comorbidities. Conventional medical treatment includes various psychotropic medications such as atypical antipsychotics, selective serotonin reuptake inhibitors (SSRIs), stimulants, and anxiolytics.^2^ However, use of these medications for ASD is often not effective and is commonly associated with adverse drug reactions.

Cannabidiol is a cannabis derivative that exhibits no addictive and neuropsychiatric properties. Hence, it is the preferred product for use in children, and, at the initiation of their parents and with support from their physicians, increasing numbers of ASD children are receiving approval for cannabis products to combat autistic symptomatology.

In this observational retrospective study our team reviewed children diagnosed with ASD and receiving their cannabis from Tikun Olam Ltd (Tel Aviv, Israel) (see below). They were administered oral oil-based cannabinoid extract drops, after permission was granted by the Israeli Ministry of Health for administration under the supervision of the children’s pediatricians. For the purpose of this retrospective review, the Shamir Hospital Research ethics committee approved an anonymous retrospective review of the patients’ charts.

The cannabinoid oil solution used was prepared by Tikun Olam Ltd (Tel Aviv, Israel) at a concentration of 30% and at 1/20 ratio of cannabidiol (CBD) and Δ9-tetrahydrocannabinol (THC). During the first meeting, parents were instructed by a nurse practitioner on how to administer the preparation. Thereafter, a biweekly follow-up telephone interview was conducted, where parents reported on effects and adverse effects of the cannabis given to their children and issues of efficacy and safety were discussed. Typically, therapy started with one or two drops given under the tongue, with the dose increased or decreased based on the child’s reported response.

Fifty-three children at a median age of 11 (range 4–22) years received cannabis and were followed up for changes in ASD-related symptoms.^3^ Median treatment duration was 66 days (range 30–588); median daily CBD and THC doses were 90 mg (range 1.5–315) and 6.75 mg (range 0.5–49.5) mg, respectively. The parents reported a notable improvement in self-injury and rage attacks, hyperactivity, sleep problems, anxiety and mood problems, and social communication and reciprocity issues (Table 1).

**Table 1:** Reported Improvement in Behavior Problems after Administration of Cannabinoid Oil Solution.^3^

Reported Problem	Number (% of cohort)	Reported Improvement, *n* (%)
Self-injury and rage	34 (64.2%)	23 (67.6%)
Hyperactivity	30 (56.6%)	21 (70.0%)
Sleep problems	21 (39.6%)	15 (71.4%)
Anxiety and mood problems	17 (32.1%)	8 (47.1%)
Social communication and reciprocity	15 (28.3%)	13 (86.7%)

When parents (51 out of 53) were asked to evaluate the overall change in their children’s ASD symptoms, significant improvement was reported in 43.1%, mild to moderate improvement was reported in 31.4%, and no change was reported in 21.6%; in the case of 2 children (3.9%), parents reported a worsening of symptoms. The most common reported adverse effects were somnolence, nausea, and change in appetite.

These results are consistent with other preliminary reports of the effectiveness of cannabis in ASD.^4^,^5^ Hence, based on the parents’ reported outcomes, this study suggests that cannabidiol may be effective in improving ASD comorbidities symptoms, with few and transient adverse effects. However, the long-term effects and safety of these preparations should be evaluated in further large-scale, controlled studies.

### Use of Cannabis in Fetal Alcohol Spectrum Disorder

Fetal alcohol spectrum disorder has been recently estimated to afflict up to 5% of American children.^6^ Most of these children exhibit different degrees of symptomatology of disruptive behaviors.^7^ Yet, there has been very little research on the efficacy and safety of pharmacological modalities, limited mostly to stimulants for attention deficit hyperactivity disorder (ADHD), or second-generation atypical antipsychotics for aggression.^8^ As shown above, recently, the use of cannabinoids has been described for symptoms related to ASD as well as for other disruptive behaviors, with apparent favorable effects.^4^ We therefore performed a retrospective pilot study to analyze the effect of cannabis in children and young adults diagnosed with FASD according to internationally accepted criteria.^9^ The study protocol was approved by the Shamir Hospital Research Ethics Committee, and informed consent was given by the parents for use of anonymous data.

All reviewed cases (three children and two young adults) had been diagnosed by us in the Motherisk Israel Program in recent years as suffering from FASD. (Motherisk is a clinical and research program at Yitzhak Shamir Medical Center in Be’er Ya’akov, Israel.) At the time of diagnosis none of the children had received cannabinoids. In all cases, the parents initiated cannabinoid use. The three children received cannabidiol, and the two young adults received THC. Disruptive symptomatology was ranked by the parents’ version of the Nisonger Child Behavior Rating Form (N-CBRF), a visual analog scale (VAS) for disruptive symptoms.^10^

Changes in disruptive symptoms before versus after cannabis use were quantified by Student’s *t* test for paired data. There was a highly statistical decrease in the disruptive behavior score in all five patients, from 18±1.0 prior to cannabis use to 6±2.1 following introduction of cannabis (*P*=0.0002).^11^

These cases suggest that the efficacy and safety of CBD should be tested in well controlled studies. Adding CBD to the very restricted armamentarium of pharmacological solutions for disruptive behavior may be very meaningful, especially because second-generation antipsychotic drugs carry a high risk of adverse central nervous system effects, in addition to serious weight gain and complex metabolic changes.

### The Use of Cannabis for Hyperemesis Gravidarum

The most severe form of nausea and vomiting of pregnancy, HG, responds only partially to standard antiemetic medications. Cannabis has been known to possess antiemetic effects, and there are several medicinal cannabinoids used as antiemetics for cancer chemotherapy. Its favorable use for HG has been described in social media, but only a few papers could be found in the medical literature.

In 2006, Westfall et al. reported on cannabis use for HG in 59 women. Of these, 40 women had used cannabis to treat their nausea; of those, 37 considered the cannabis to have been “extremely effective” or “effective” in controlling their symptoms.^12^

Hence, we evaluated four Motherisk-counseled cases of HG, following cannabis use. There was a highly significant improvement in symptoms: the validated Pregnancy-Unique Quantification of Emesis (PUQE)^13^ score improved from 14.5±1 to 7.5±0.58 (*P*=0.0004). Cannabis use was associated with a significant increase in PUQE Quality of Life scale, from 2±0.82 to 7±0.82 (*P*=0.0012).^14^

Based on these four cases, cannabis appears to be an effective drug for HG, and should be further researched in controlled studies. Presently, with the legalization of cannabis in the USA and other countries, there are numerous reports on social media of women describing dramatic therapeutic effects. However, this four-case series is the first academic report with objective and validated measures of response.

Nevertheless, Metz and Stickrath’s literature review reveals an ongoing concern regarding the use of cannabis in pregnancy due to modern marijuana’s high Δ9-tetrahydrocannabinol content, which crosses the placental barrier and can lead to smaller birth weights, and still and pre-term births.^15^

## DISCUSSION

Herein we described the retrospective cannabis experience in three disease entities, which to date are lacking in effective pharmacological modalities. The observations from these studies have reflected dramatic results; however, these preliminary series are limited by small numbers that preclude any definitive therapeutic or safety conclusions. While this review does not offer the methodological rigor needed to approve cannabis use in children and pregnant women, it does provide important clues to inform the medical community regarding the possible design and execution of future definitive studies.

In the case of HG, there are concerns that cannabis may disrupt the normal trajectory of fetal brain development; however, scores of studies following children whose mothers had used cannabis recreationally have failed to show consistent effects.^16^

In all three instances described herein, cannabis use was initiated by the parents due to frustration with the lack of initiative by the medical scientific community. Medical cannabis usage in children and pregnant women needs to be thoroughly discussed and researched by the academic community, so that it can be applied appropriately and in the most effective manner in these patient populations.

## Abbreviations

ASD: autism spectrum disorder
CBD: cannabidiol
FASD: fetal alcohol spectrum disorder
HG: hyperemesis gravidarum
SSRI: selective serotonin reuptake inhibitors
THC: tetrahydrocannabinol.
